# Hypertension Exacerbates Endothelial Dysfunction in Patients With Atrial Fibrillation

**DOI:** 10.1111/jch.70028

**Published:** 2025-04-06

**Authors:** Samuel Thomas, Fiona L. Wilkinson, Amy R. Bland, Gregory Y. H. Lip, James P. Fisher, Rehan T. Junejo

**Affiliations:** ^1^ Department of Life Sciences Faculty of Science and Engineering Manchester Metropolitan University Manchester UK; ^2^ School of Psychology Faculty of Health and Education Manchester Metropolitan University Manchester UK; ^3^ Liverpool Centre for Cardiovascular Science at University of Liverpool Liverpool John Moores University and Liverpool Heart & Chest Hospital Liverpool UK; ^4^ Danish Center for Health Services Research Department of Clinical Medicine Aalborg University Aalborg Denmark; ^5^ Faculty of Medical and Health Sciences Department of Physiology University of Auckland Auckland New Zealand

**Keywords:** atrial fibrillation, flow‐mediated dilatation, hypertension, shear stress

## Abstract

Atrial fibrillation (AF) and hypertension (HT) often coincide and both are independently associated with endothelial dysfunction. We tested the hypothesis that brachial artery flow‐mediated dilation (FMD), an indicator of endothelial health, will be poorer in AF patients with HT (AF + HT) than AF without concurrent HT. In a cross‐sectional design study, AF (*n* = 29; mean 70 years; 9 females) and AF + HT (*n* = 33; 68 years (*p *= 0.302); 14 females) patients underwent Duplex‐Doppler ultrasound imaging of brachial artery diameter and flow velocity during baseline (2 min), distal tourniquet cuff inflation (5 min), and following cuff deflation (3 min). The peak increase in artery diameter following cuff deflation was taken as FMD and analyzed as absolute, percentage change, FMD and shear‐rate area‐under‐the‐curve (SR_AUC_; FMD‐to‐SR_AUC_) ratio, and using SR_AUC_ as a covariate (FMD_SRAUC_). Body mass index (BMI) was used as an additional covariate for between‐group comparisons of vascular data. Mean arterial pressure was higher in the AF + HT versus the AF group (median [interquartile range] 93 [85–99] vs. 84 [80–90] mm Hg, respectively; *p *< 0.05). Baseline brachial artery diameters were similar (*p *> 0.05). FMD was lower in AF + HT than AF patients (3.36 [1.69–5.21] vs. 4.98 [2.96–7.11] %, respectively; *p *< 0.05). Similar group differences were observed in absolute FMD, FMD‐to‐SR_AUC_ ratio and FMD_SRAUC_ (*p* < 0.05). AF patients with concurrent HT exhibit poorer endothelium‐dependent vasodilation compared to AF patients, indicating that the presence of comorbid HT exacerbates endothelial dysfunction in AF patients.

## 1 Introduction

Atrial fibrillation (AF) is the most common sustained arrhythmia disorder worldwide and continues to be of clinical significance as a global health burden [1]. The global prevalence of AF is set to increase due to improved survival from previously fatal cardiovascular events and better diagnostic tools. Hypertension (HT) is the most common modifiable risk factor and cardiovascular disease to coexist in AF patients [2]. HT is also thought to be responsible for 50% of all AF diagnoses and accounts for more AF cases than any other risk factor [3]. Although AF and HT are independently linked with adverse clinical events, the simultaneous occurrence of both conditions has been linked to worsened clinical prognosis than either alone [4].

The endothelial lining of the blood vessels provides a single‐cell mechano‐ and metabosensitive paracrine interface between the bloodstream and vascular wall. The health of vascular endothelium plays a key role in regulating fluid filtration, platelet activation, inflammation, cellular proliferation, and vascular tone [5]. The importance of endothelial health is well established with endothelial dysfunction reported in cardiovascular diseases such as coronary artery disease, HT, renovascular disease, and AF [5].

In AF, the irregular heart rhythm and resultant turbulent flow results in an erratic and low‐shear stress state, which promotes endothelial dysfunction [6]. Indeed, endothelium‐dependent flow‐mediated dilation (FMD) is attenuated in AF patients alongside an increase in the plasma biomarker of endothelial damage/dysfunction, von Willebrand factor [7]. Separately, increased pulsatility and blood pressure (BP) associated with HT promotes elevated vascular oxidative stress and inflammation, which hold a central role in the development of endothelial dysfunction and leads to decreased production and bioavailability of nitric oxide (NO) [8, 9].

Although independent evidence of endothelial dysfunction is present for AF and HT, it remains unclear whether the simultaneous presence of comorbid HT in AF patients (AF + HT) exacerbates endothelial dysfunction. Using brachial artery ultrasound imaging for the assessment of FMD, a consistently reliable indicator of overall endothelial and generalized cardiovascular health [10], herein we investigate whether AF patients with HT (AF + HT) exhibit a worsened brachial artery FMD compared to lone AF patients. We tested the hypothesis that AF + HT patients will present with worse FMD compared to AF patients without HT.

## 2 Methods

### 2.1 Ethical Approval

All implemented procedures conformed with the Declaration of Helsinki and were approved by the National Research Ethics Service Committee Northwest (17/NW/0714). Funding support was supplied by the Bristol‐Myers Squibb‐Pfizer Alliance. Potential participants were informed of study procedures through a verbal explanation and an information sheet. Written informed consent was obtained prior to procedures.

### 2.2 Participant Characteristics

Sixty‐two AF patients from our previous study investigating the impact of Warfarin and Apixaban on vascular health [11] who fulfilled the inclusion criteria of the present study were separated into two groups: AF without HT (*n* = 29) and AF with HT (*n* = 33; Table 1). Recruitment was carried out from City Hospital, Birmingham; Liverpool Heart and Chest Hospital, Liverpool; and West Midlands GP practices within the National Institute of Health Research Clinical Research Network. Exclusion criteria included current smokers, ventricular dysfunction, myocardial infarction, premenopausal women, hepatic, connective tissue, respiratory, neurological, inflammatory, and malignant diseases, stroke or transient ischemic attack (<3 years), and valvular heart disease. Patients had received a clinical diagnosis of at least paroxysmal AF without or with primary HT. A list of medications being consumed by patients is presented in Table S1.

**TABLE 1:** Participants characteristics of AF and AF + HT patients.

	AF	AF + HT	*p* value (*d)*)
*N*	29	33	
Females *n*, %	9, 31	14, 42	0.354
Age (years)	70 ± 8	68 ± 7	0.302
Height (cm)	172 ± 9	171 ± 11	0.681
Weight (kg)	79 [70–85]	90 [77–98]	0.040 (0.557)
BMI (kg/m^2^)	26 [24–30]	30 [28–31]	0.002 (0.807)
Waist circumference (cm)	93 [84–101]	96 [93–104]	0.022 (0.667)
Hip circumference (cm)	102 [98–107]	109 [105–115]	<0.001 (0.965)
Waist/height ratio (au)	0.54 ± 0.06	0.58 ± 0.06	0.003 (0.790)
Waist/hip ratio (au)	0.90 ± 0.08	0.91 ± 0.09	0.587
Heart rate (b/min)	68 [60–70]	68 [62–80]	0.258
Systolic BP (mm Hg)	137 ± 15	150 ± 14	<0.001 (0.977)
Diastolic BP (mm Hg)	80 [75–85]	88 [80–94]	0.005 (0.718)
Mean BP (mm Hg)	84 [80–90]	93 [85–99]	0.001 (0.806)
Stroke volume (mL)	64 [47–94]	67 [51–85]	0.860
Cardiac output (L/min)	4 [2–6]	4 [4–6]	0.309
TPR (mm Hg·s/mL)	1.25 [0.81–1.90]	1.31 [0.94–1.70]	0.865
CHA_2_DS_2_VASc	2 [1–2]	3 [2–3]	0.007(0.668)
Years since AF diagnosis	5 [3–9]	5 [2–12]	0.786
Paroxysmal AF *n*, %	16, 55	15, 45	0.427
Persistent AF *n*, %	13, 45	17, 52	0.517
Permanent AF *n*, %	0, 0	1, 3	0.345
Weekly alcohol intake (units)	2.00 [0.00–10.00]	4.00 [1.00–10.00]	0.654

*Notes*: Values expressed as the mean ± SD for normally distributed data, as median [interquartile range] for non‐normally distributed data, and as frequency (percentage) for discrete/categorical variables. Statistical differences were tested using an independent *t* test or Mann–Whitney *U* test as appropriate for continuous variables and using Pearson's Chi‐squared test for categorical variables. If significant, Cohen's *d* or Phi were reported for continuous and categorical variables, respectively. Significance: *p* < 0.05.

Abbreviations: BMI, body mass index; BP, blood pressure; *d*, Cohen's *d*; TPR, total peripheral resistance.

### 2.3 Experimental Measures

Medication use, current alcohol use, and medical history were recorded. Medical history was used to calculate participant CHA_2_DS_2_‐VASc scores. Anthropometric measures such as height, weight, waist (at the level of umbilicus), and hip (at the level of femoral trochanter) circumference were obtained. The brachial artery was assessed with an automated oscillometric device (M2, Omron, Kyoto, Japan) for BP and heart rate (HR) measurements. Finger photoplethysmography was used to collect 10‐min supine beat‐by‐beat measures of BP (Finometer MIDI, Finapres Medical Systems, Amsterdam, the Netherlands). Subsequently, right brachial artery diameter and flow velocity measurements were simultaneously captured while the arm was supported at the heart level using a Duplex Doppler ultrasound (GE LOGIQe, GE Healthcare China Corporation, Jiangsu, China). Insonation of the brachial artery occurred at 60° proximal to the medial epicondyle. Imaging of vessel diameter and the pulse wave mode used for assessing blood flow velocity was performed using the B‐mode on a 5–13 MHz multi‐frequency linear‐array transducer (GE 12L‐RS, GE Healthcare Japan Corporation, Tokyo, Japan). Video files were recorded and stored for offline analysis utilizing an automated vessel wall tracking software (Cardiovascular Suite Version 3.4.1, FMD studio, Pisa, Italy). Vascular assessments were performed in compliance with the recent technical recommendations [12].

### 2.4 Experimental Protocol

Prior to testing, patients were asked to avoid vigorous exercise, food, and caffeine for 12 h and alcohol for 24 h. On the day of the study visit, patients were required to abstain from taking usual morning medications with the exception of anti‐coagulants. Following consent, questionnaires and anthropometric measurements, patients were required to lie in a supine position on a medical examination couch. Resting measurements of brachial artery BP and HR were taken three times ∼2 min apart from one another and then averaged. Ten minutes of resting supine beat‐by‐beat BP recordings were than collected with the photoplethysmograph cuff wrapped around the middle or ring finger. An inflatable tourniquet cuff (Hokanson, Bellevue, WA, USA) was then placed around the widest part of the forearm. Brachial artery was insonated for diameter and pulse‐wave before the FMD protocol was implemented as follows: a 2 min baseline, followed by a 5 min supra‐systolic cuff inflation (>240 mm Hg), finished by 3 min recovery.

### 2.5 Data Analysis

Calculations for waist‐to‐hip and waist‐to‐height ratios were done in addition to body mass index (ratio of weight‐to‐height squared). Mean BP was calculated as diastolic BP (mm Hg) + [0.33 + (HR × 0.0012)] × [systolic BP (mm Hg) − diastolic BP (mm Hg)] [13]. Finger photoplethysmography pressure waves were also utilized to calculate stroke volume (SV), cardiac output (CO), and total peripheral resistance (TPR) using the Windkessel Model (non‐invasive cardiac output feature on LabChart v 8.1.19, ADInstruments). For brachial artery measurements, the baseline period was computed as a 2‐min average and on second‐by‐second basis amid the 3 min of recovery following cuff deflation. The maximal increase in brachial artery diameter compared to baseline diameter was determined as FMD and expressed as absolute (mm) and relative (%) change. The time between cuff deflation and the maximal artery dilation was measured in seconds for time‐to‐peak. The calculation for the shear rate (SR) was as follows:

$$\mathrm{Shear}\;\mathrm{rate}\;=\frac{\mathrm{Brachial}\;\mathrm{artery}\;\mathrm{flow}\;\mathrm{velocity}\cdot 4}{\mathrm{Brachial}\;\mathrm{artery}\;\mathrm{diameter}}$$

The shear rate area under the curve (SR_AUC_) was determined by calculating the integral between cuff deflation to the point of maximal artery dilation. In compliance with published guidance [12], SR_AUC_ was implemented as a covariate for the calculation of SR‐corrected FMD (FMD_SRAUC_). Additionally, the FMD‐to‐SR_AUC_ ratio was also calculated and multiplied by 1000 [14, 15]. Further, slope and upper bound 95% confidence intervals of the log‐transformed baseline and peak diameters were calculated following the guidelines for allometric scaling of baseline artery diameters [16]. As the slope (0.99) and 95% confidence intervals (1.05) were close to unity, allometric scaling was not performed. Brachial velocity profiles reported are of net velocity (positive minus negative velocity). The time between cuff deflation to peak net brachial velocity was measured to determine time‐to‐peak velocity. Brachial artery blood flow (mL/min) was calculated as follows:

$$\begin{array}{ccc} & & \mathrm{Brachial}\;\mathrm{artery}\;\mathrm{blood}\;\mathrm{flow}\;\\ & & \;=\;\left[\frac{\mathrm{Peak}\;\mathrm{envelope}\;\mathrm{velocity}}{2}\cdot \left(\pi \;{\left(0.5\;\cdot \;\mathrm{Diameter}\right)}^{2}\right)\right]\;\cdot 60 \end{array}$$

Peak brachial artery blood flow was obtained and taken as peak hyperemia. Time‐to‐peak hyperemia was determined as the time from cuff deflation to peak blood flow response.

### 2.6 Statistical Analysis

**FIGURE 1:**
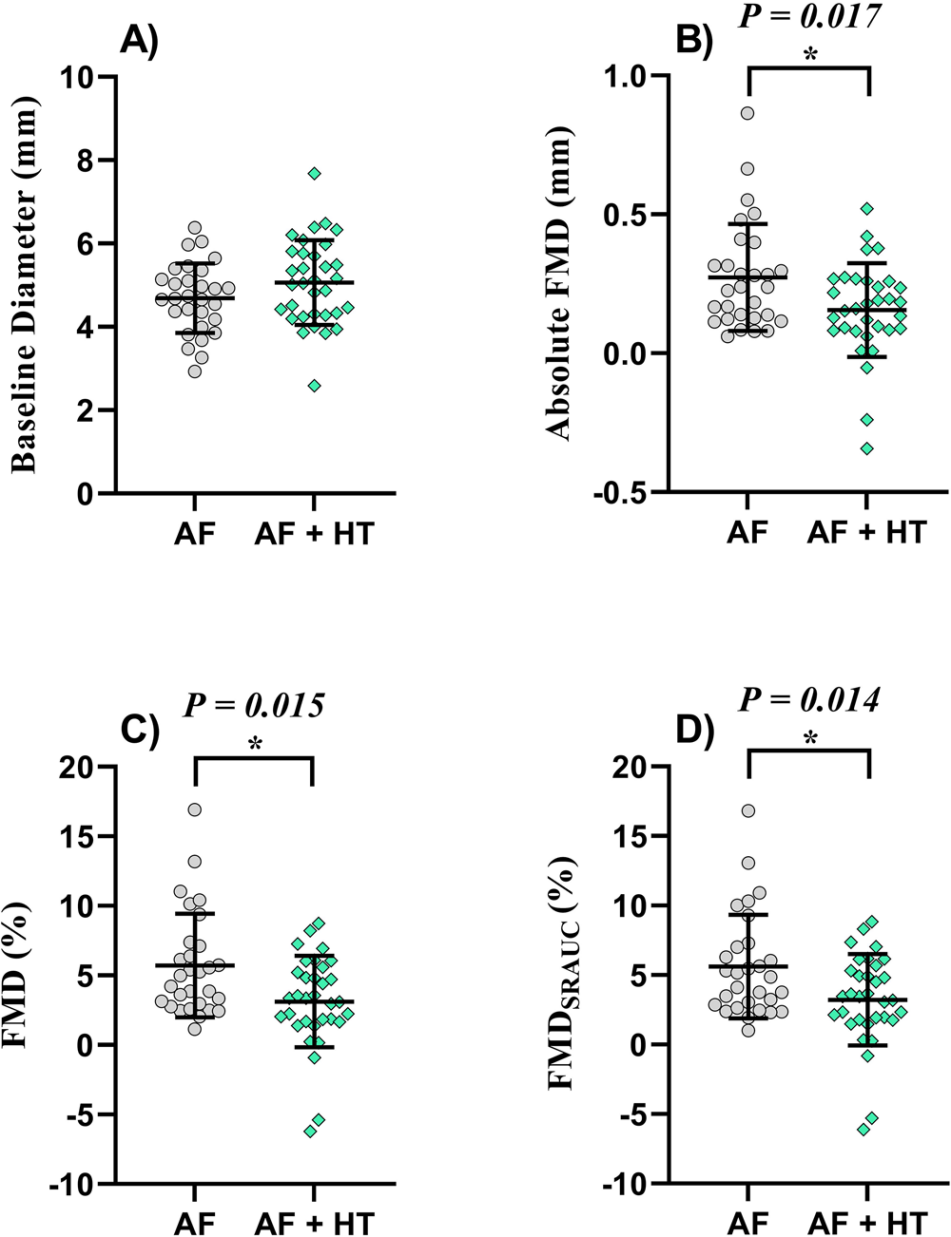
Baseline brachial artery diameter (A), absolute FMD (B), FMD (C), and FMD_SRAUC_ (D) in participants with AF and AF + HT. Individual values are plotted with bars displaying mean and standard deviation. AF, atrial fibrillation; AF + HT, atrial fibrillation with hypertension; FMD_SRAUC_, shear rate area under the curve as covariate corrected flow‐mediated dilatation; FMD, flow‐mediated dilation.

The analysis was performed using IBM SPSS Statistics, version 29 (IBM Corp., Armonk, NY, USA). Continuous variables were all tested for normality using a Shapiro–Wilk test. Variables determined to be not normally distributed were transformed using the natural logarithm method. An independent two‐tailed Student's *t*‐test was used for the analysis of normally distributed data presented in Table 1. Meanwhile, a Mann–Whitney *U* test was utilized for comparison when data continued to remain not normally distributed. Pearson *χ* ^2^ was used for analysis of categorical data. Group differences were observed for weight, body mass index (BMI), waist, and hip circumferences as well as waist to height ratios (Table 1). Due to the high degree of significant (*r* > 0.84; *r* ^2^ > 0.70; *p* < 0.001) correlation observed between these variables, BMI was chosen as a covariate in a one‐way analysis of covariance (ANCOVA) for further group comparisons and analysis (Table 2 and Figure 1). FMD_SRAUC_ analysis had SR_AUC_ and BMI as covariates. Cohen's *d* (*d*) and Phi values (*φ*) were reported for significant continuous and dichotomous/categorical measures, respectively. Transformed values were used for *d* calculations when statistical comparisons were performed on log‐transformed data. Continuous and dichotomous variables presented in Tables 1 and S1 (with >5 data points) were entered into a stepwise multiple regression to identify their effects on brachial FMD (log‐transformed). To reduce the risk of regression model violations, multi‐collinearity and autocorrelations were calculated. Tolerance of the reported variables was 1.00, the variance inflation factor was also 1.00 and the Durbin–Watson statistic was 2.297. Presented data are expressed as either mean ± standard deviation (SD) for normally distributed data, median [interquartile range] for not‐normally distributed data or as frequency (percentage) for categorical measurements. Data are presented as original. Log‐transformed data used for analysis were reverse‐transformed before being reported.

**TABLE 2:** Flow mediated dilatation and reactive hyperemia characteristics in AF and AF + HT patients.

	AF	AF + HT	*p* value (*d*)
Baseline diameter (mm)	4.69 ± 0.84	5.06 ± 1.02	0.244
Baseline velocity (cm/s)	8.55 [6–13.73]	9.35 [5.66–12.20]	0.278
Baseline blood flow (mL/min)	47.41 [27.74–69.44]	52.62 [33.90–76.66]	0.709
∆ velocity (cm/s)	64.50 ± 17.75	61.87 ± 17.21	0.492
Peak velocity (cm/s)	75.77 ± 19.83	71.77 ± 18.68	0.253
Time to peak velocity (s)	10 [9–13]	9 [7–11]	0.506
∆ blood flow (mL/min)	309.29 [207.00–430.51]	377.67 [224.79–425.36]	0.739
Peak hyperemia (mL/min)	369.92 [259.46–469.42]	433.77 [268.59–526.82]	0.865
Time to peak hyperemia (s)	10 [8–14]	9 [6–11]	0.235
SR_AUC_ (/s)	14659.60 [9982.80 ‐17116.20]	11517.00 [9652.20–14315.70]	0.460
Peak diameter (mm)	4.96 ± 0.93	5.22 ± 1.05	0.563
Time to peak diameter (s)	68 ± 27	64 ± 31	0.707
Absolute FMD (mm)	0.24 [0.13–0.32]	0.16 [0.08–0.26]	0.017 (0.501)
FMD (%)	4.98 [2.96–7.11]	3.36 [1.69–5.21]	0.015 (0.480)
FMD_SRAUC_ (%)	4.87 [2.85–7.00]	3.46 [1.79–5.31]	0.014 (0.530)
FMD‐to‐SR_AUC_ ratio (au)	0.33 [0.24–0.56]	0.30 [0.15–0.46]	0.035 (0.407)

*Notes*: Values are displayed as mean (SD) when normally distributed or median [interquartile range] when non‐normally distributed. A one‐way ANCOVA was used to determine statistical differences using BMI as a covariate; not‐normally distributed data were log‐transformed prior to analysis.

Abbreviations: ∆, change from baseline; *d*, Cohen's *d*; FMD, flow‐mediated dilatation; FMD_SRAUC_, shear rate area‐under‐the‐curve as covariate corrected flow‐mediated dilatation; FMD‐to‐SR_AUC_, flow‐mediated dilation to shear rate area‐under‐the‐curve ratio; SR_AUC_, shear rate area‐under‐the‐curve.

## 3 Results

### 3.1 Participant Characteristics

Tables 1 and S1 summarize participant characteristics and medication use, respectively. There was no difference in age or sex between groups. For anthropometric measurements, the AF + HT group weighed more than AF (Median [IQR] 90 [90–98] vs. 79 [70–85] kg, respectively; *p *= 0.040; Table 1), while BMI (*p *< 0.001), waist (*p* < 0.05), and hip circumferences (*p* < 0.001) were also greater in AF + HT group. Height and waist‐to‐hip ratio were not different between groups (Table 1). As expected, resting systolic, diastolic, and mean BP were greater in the AF + HT versus the AF group (*p* < 0.001; Table 1). No differences were observed for HR, CO, SV, and TPR between both groups (Table 1) showing groups were hemodynamically well matched. AF + HT patients had a significantly higher CHA_2_DS_2_VASc risk score than AF (*p* < 0.05). There were no significant differences in time since AF diagnosis, type of AF, or weekly alcohol intake (*p* > 0.05). For medication use, as expected AF + HT patients had a greater use of antihypertensive ACE inhibitors, calcium channel blockers, and angiotensin receptor blockers (*p* < 0.001; Table S1).

### 3.2 Flow Mediated Dilation

There were no significant differences in baseline diameter, peak diameter, time‐to‐peak diameter, and SR_AUC_ between the groups (*p* > 0.05; Table 2). The AF group exhibited a greater absolute FMD (0.24 [0.13–0.32] vs. 0.16 [0.08–0.26] mm; *p *= 0.017) and percentage FMD response (4.98 [2.96–7.11] vs. 3.36 [1.69–5.21]%; *p *= 0.015) compared to AF + HT patients (Figure 1). FMD‐to‐SR_AUC_ ratio (0.33 [0.24–0.56] vs. 0.30 [0.15–0.46] au; *p *= 0.035) and ANCOVA corrected FMD_SRAUC_ responses (4.87 [2.85–7.00] vs. 3.64 [1.79–5.31]%; *p* = 0.014; Table 2) were also greater in AF compared to AF + HT patients. FMD responses were not different between AF + HT patients with resting BP higher than or below 140/90 mm Hg (*p* > 0.11).

### 3.3 Reactive Hyperemia

There were no differences in baseline velocity, peak velocity, ∆ change in velocity, or time‐to‐peak velocity (*p* > 0.05; Table 2). Baseline blood flow, peak blood flow, and ∆ change in blood flow were also similar between groups (*p* > 0.05). Time‐to‐peak hyperemia (*p *= 0.235) was also not different between groups.

### 3.4 Stepwise Regression

CHA_2_DS_2_VASc score predicted brachial FMD response (F (1,55) = 4.826, *p* = 0.032; Table 3) and was negatively correlated by 0.85% and accounted for ~8% of the variation in outcome variable data.

**TABLE 3:** Stepwise multiple regression with FMD (%) as the dependent variable.

	*β*	*B*	95% CI for *B*	*p* value
Dependent variable: FMD (%) *R* = 0.284; *R* ^2^ = 0.081; Adjusted *R* ^2^ = 0.064; *p* = 0.032				
CHA_2_DS_2_VASc	−0.284	−0.158	−0.302 to −0.014	0.032

*Notes*: Variables reported in Tables 1 and S1 were entered into a stepwise multiple regression with a log‐transformed FMD (%) as the dependent variable. Independent variables with ≤5 data points were excluded from the model. The Tolerance for the reported variable was 1.000, the variance inflation factor was also 1.000, and Durbin–Watson was 2.297.

Abbreviations: *β*, standardized coefficient; *B*, unstandardized coefficient; CI, confidence interval; *R*, correlation coefficient; *R^2^*, coefficient of determination.

## 4 Discussion

As far as we are aware, this is the first study to explore whether the presence of HT in AF patients further worsens peripheral endothelial function. We observed that shear stress‐driven brachial artery FMD, a well‐established indicator of endothelial function, was poorer in AF patients with HT compared to AF patients without HT. These findings provide the scientific rationale and may explain the increased risk of adverse cardiovascular events in AF patients who present with comorbid HT [4, 17, 18].

Adult HT is often a silent disease [19] and untreated/poorly‐treated HT increases the risk of all cause and cardiovascular mortality [20]. Comorbid HT is often reported in AF and is recognized as a major contributor to the development of AF^2^. HT promotes the emergence of electrophysiologic arrhythmias as a consequence of hemodynamic alterations, neuroendocrine elements, hypertrophy of the left ventricle, and structural modifications to both the atria and ventricles due to myocardial fibrosis [21]. Additionally, the persistent elevation of BP in HT induces mechanical overload, resulting in myocardial changes and atrial cardiomyopathy [22]. The prognostic information gathered from FMD is of clinical relevance and reliably predicts future adverse cardiovascular events [23].

Independently AF and HT are known to exhibit impaired FMD. For example, prior studies have independently reported a reduced FMD response and greater release of von Willebrand factor in AF patients compared to healthy controls [7, 24, 25]. Separately, impaired FMD and higher circulating concentrations of von Willebrand factor have been noted in HT patients compared to healthy controls [26]. However, this does not necessarily equate to an additive endothelial dysfunction if the clinical conditions present as comorbidities. Indeed, our previous work [27] explored endothelial function in an AF + HT population, but unlike this study, compared them to a lone HT patient group. A similar FMD response previously found between HT and AF + HT patients suggested that AF development in HT patients may be of limited consequence for endothelial health. However, we did note that the duration and frequency of AF were strong predictors of attenuated FMD rather than presence of AF per se.

Although comorbid HT is present in several AF patients, a significant number of patients do present to the clinics without HT. Polovina et al. [24] and Heshmat‐Ghahdarijani et al. [25] have previously observed attenuated brachial artery FMD in such AF patients compared to healthy controls. Herein, whilst controlling for the duration and frequency of AF (i.e., similar time of diagnosis and type of AF) and for the cardiac hemodynamics (i.e., similar stroke volume and cardiac output), our novel findings of attenuated absolute FMD, percentage FMD, FMD_SRAUC_, and FMD‐to‐SR_AUC_ ratio strongly implicate HT as an important comorbid condition, revealing its unique additive effects on endothelial dysfunction in AF patients. Importantly, when AF + HT patients were split into those presenting with resting BP responses higher than or lower than 140/90 mm Hg on testing day, no significant differences were observed for any of the FMD responses between them. However, this must be considered under the observation that the resting BP measurement herein reflects a spot measurement on testing day rather than overall day‐to‐day BP control of HT patients.

Our recent novel findings may in part be explained by the HT‐evoked vascular remodeling, wall thickening, and arterial stiffness [28]. In HT, oxidative stress is also an important relevant factor, driving damage to the cellular macromolecules, contributes to NO quenching, and vascular smooth muscle proliferation/remodeling [29, 30]. Furthermore, elevated oxidative stress can drive inflammation and vice versa, which are both associated with vascular aging [30]. Elevation in the levels of interleukin‐6 and tumor necrosis factor‐α and activation of circulating levels of T cells and B cells have all been found in (and linked with) HT development [31, 32]. Moreover, the high pressure within circulation also drives extracellular matrix deposition within the vessel walls [33]. We have previously observed a trend toward improved FMD in HT patients following 8 weeks of BP medication optimization [27]. There are reports suggesting HT itself does not reduce the internal wall diameter of conduit muscular arteries [34]. In agreement, we have also failed to observe differences in baseline brachial artery diameter between our AF and AF + HT patients. Nonetheless, reports of oxidative stress and vascular inflammation‐driven endothelial dysfunction in HT may be of relevance to explain our FMD findings. Levels of antioxidant scavengers such as superoxide dismutase and glutathione peroxidase are decreased in HT [35, 36] whereas ascorbic acid and vitamin E supplementation have been found to restore impaired endothelium‐dependent vasodilation and reduce central pulse wave velocity in HT patients [37].

Attenuated FMD, revealing endothelial dysfunction, is an independent predictor of future adverse cardiovascular events [23]. Our findings of attenuated FMD in AF + HT compared to AF have important implications for patient care and management. Combined with the observations of substantially increased stroke risk and attenuated cerebrovascular vasodilatory reserve in AF patients who present with comorbid HT [17, 18, 38], our novel findings provide the scientific and mechanistic rationale for the argument of careful/optimal BP control in AF patients who present with comorbid HT.

In our stepwise regression, CHA_2_DS_2_VASc was a significant negative predictor of FMD accounting for ∼8% of the variation in data. CHA_2_DS_2_VASc was significantly different between groups. This outcome is unsurprising as the presence of HT is one of the factors contributing to the CHA_2_DS_2_VASc risk score [39]. Previous observations have associated low FMD and greater CHA_2_DS_2_VASc scores with increased risk of adverse cardiovascular events [40].

Our study findings should be considered in the light of following considerations. Screening for molecular and cellular risk factors were beyond the remit of this study. Although the approach of requesting abstinence from medications on study morning is unlikely to entirely abrogate the vasoactive impact of medications, this approach was adopted to at least control for their acute influence on recorded data whilst keeping patient welfare under consideration. Allowance of anticoagulants to AF patients was for the same ethical reason. The relationship between obesity and endothelial dysfunction is not straight‐forward. Although some have observed attenuated endothelial function [41, 42], raised markers of oxidative stress/inflammation [43] and increased risk of cardiovascular disease [44, 45], others have suggested the presence of metabolically healthy obesity amongst certain individuals [46]. Given the evidence of obesity and body fat distribution‐related endothelial dysfunction [47], it is a possibility that some of our vascular data reflect this. To control for these factors affecting our vascular/FMD comparisons between AF and AF + HT patients, the statistical approach of ANCOVA was employed where BMI was added to all vascular analysis as a covariate. Exclusion of weight, waist, and hip circumference as covariates was determined by high degree of statistically significant (*p* < 0.001) correlation between these variables. Covariate‐controlled statistical analysis findings support our conclusions. Although the time since disease diagnosis and the type of AF present at the time of study were not different, it is near impossible to accurately determine the frequency of AF episodes. Moreover, given the silent nature of HT an accurate timeline for its development is also near impossible. HT‐driven AF can take years to manifest [48, 49]. The majority of patients were recruited from dedicated AF clinics at hospitals and as HT patients often could not remember the duration since their HT diagnosis, this was not recorded. We have reported the use of medication and found no differences between groups except for obvious antihypertensive medications. Nonetheless, future studies should aim to also collect a record of previous treatments and timeline of all comorbidities, if possible. It is also possible that HT disease of a shorter duration and/or reduced severity may have limited adverse impact on endothelial health of AF + HT patients. This idea should be explored in future studies. Blood samples were not collected; therefore, no correlation could be made between the level of endothelial dysfunction and blood biomarkers of endothelial health, oxidative stress, and inflammation. Finally, future studies should consider direct comparisons between AF, HT, and AF + HT patients.

In conclusion, this study provides novel findings that AF patients with HT display worse endothelial dysfunction (attenuated FMD) compared to AF patients without HT. Given attenuated FMD provides reliable prediction of future adverse cardiovascular events, clinical professionals are advised to take the presence and appropriate management of HT into account to reduce the risk of additional adverse events in AF patients.

## Author Contributions

J.P.F., G.Y.H.L., and R.T.J.: conception. R.T.J.: data collection. S.T.: blinded data extraction, analysis, and draft of manuscript. S.T., F.W., A.R.B., G.Y.H.L., J.P.F., and R.T.J.: critique, revision and approval of final manuscript.

## Conflicts of Interest

Gregory Y. H. Lip is a consultant for Bayer/Janssen, BMS/Pfizer, Medtronic, Boehringer Ingelheim, Novartis, Verseon, and Daiichi‐Sankyo and is a speaker for Bayer, BMS/Pfizer, Medtronic, Boehringer Ingelheim, and DaiichiSankyo. No fees are directly received personally. Further conflicts of interest are not present for authors.

## Supporting information



Supporting Information

## Data Availability

Data are available from the corresponding author upon reasonable request.
