# Supplementary material for: Hypertension Exacerbates Endothelial Dysfunction in Patients With Atrial Fibrillation
Source: J Clin Hypertens (Greenwich). 2025 Apr 6;27(4):e70028. doi: 10.1111/jch.70028 (PMC11973128; doi:10.1111/jch.70028)
Supplement: Supplementary file 1 — Supporting Information [file JCH-27-e70028-s001.docx]

**Supplementary Table 1:** Medications use of AF and AF+HT patients.

| **Medication use** | **AF** | **AF + HT** | **p Value** (*φ)* |
| --- | --- | --- | --- |
| Factor Xa inhibitor | 21, 72 | 19, 58 | 0.223 |
| Vitamin K Antagonist | 8, 28 | 14, 42 | 0.223 |
| Antiplatelet | 1, 3 | 0, 0 | 0.282 |
| Alpha Blocker | 2, 7 | 1, 3 | 0.479 |
| Beta Blockers | 15, 52 | 24, 73 | 0.088 |
| Cardiac glycoside | 4, 14 | 5, 15 | 0.880 |
| Anti-arrhythmic | 6, 21 | 2, 6 | 0.086 |
| ACE Inhibitor | 0,0 | 16, 48 | <0.001 (0.553) |
| Calcium channel blocker | 0, 0 | 10, 30 | 0.001 (0.411) |
| Angiotensin receptor blockers | 0, 0 | 10, 30 | 0.001 (0.411) |
| Loop Diuretic | 0, 0 | 3, 9 | 0.096 |
| Biguanide | 1, 3 | 1, 3 | 0.926 |
| Insulin | 0, 0 | 1, 3 | 0.345 |
| PPI | 5, 17 | 11, 33 | 0.149 |
| Statins | 12, 41 | 18, 55 | 0.301 |
| Opioids | 1, 3 | 1, 3 | 0.926 |
| Long-acting beta-agonist | 0, 0 | 1, 3 | 0.345 |
| Beta 2 agonists | 1, 3 | 1, 3 | 0.926 |
| SSRI | 2, 7 | 1, 3 | 0.479 |
| Tricyclic antidepressant | 0, 0 | 2, 6 | 0.178 |
| Sulfasalazine | 1, 3 | 0, 0 | 0.282 |
| NSAIDs | 5, 17 | 2, 6 | 0.165 |
| Xanthine Oxidase Inhibitor | 0, 0 | 1, 3 | 0.345 |
| 5-alpha reductase inhibitor | 1, 3 | 1, 3 | 0.926 |
| Thyroxine | 1, 3 | 3, 9 | 0.367 |
| Musculotropic antispasmodic | 0, 0 | 1, 3 | 0.345 |
| Anticonvulsants | 1, 3 | 3, 9 | 0.367 |
| Muscarinic antagonist | 2, 7 | 2, 6 | 0.894 |
| Vitamin D | 1, 3 | 2, 6 | 0.632 |
| Anti-histamine | 1, 3 | 0, 0 | 0.282 |
| Sedative | 0, 0 | 1, 3 | 0.345 |

Values expressed as frequency, percentage for discrete/categorical variables. Group differences were tested using Pearson’s Chi-squared test. If significant, Phi values were reported. Significance: *p* < 0.05. *φ*, Phi; ACE, angiotensin-converting enzyme; PPI, Proton pump inhibitors; SSRI, Selective serotonin reuptake inhibitors; NSAIDs, Nonsteroidal anti-inflammatory drug.
